# Dr. Samuel H. Linn

**Published:** 1916-04

**Authors:** 


**Dr. Samuel H. Linn,** University of Penn'a, 1877, for some years a resident of Rochester, died early in March, aged 72.

